# The impact of software and criteria on the selection of best-fit nucleotide substitution models for molecular evolutionary genetic analysis

**DOI:** 10.1371/journal.pone.0319774

**Published:** 2025-03-26

**Authors:** Xingguang Li, Olayinka Sunday Okoh, Nídia Sequeira Trovão

**Affiliations:** 1 Ningbo No. 2 Hospital, Ningbo, China; 2 Guoke Ningbo Life Science and Health Industry Research Institute, Ningbo, China; 3 Department of Chemical Sciences, Anchor University, Lagos, Nigeria; 4 Division of International Epidemiology and Population Studies, Fogarty International Center, National Institutes of Health, Bethesda, Maryland, United States of America; Griffith University, AUSTRALIA

## Abstract

The statistical selection of best-fit models of nucleotide substitution for multiple sequence alignments (MSAs) is routine in phylogenetics. Our analysis of model selection across three widely used phylogenetic programs (jModelTest2, ModelTest-NG, and IQ-TREE) demonstrated that the choice of program did not significantly affect the ability to accurately identify the true nucleotide substitution model. This finding indicates that researchers can confidently rely on any of these programs for model selection, as they offer comparable accuracy without substantial differences. However, our results underscore the critical impact of the information criterion chosen for model selection. BIC consistently outperformed both AIC and AICc in accurately identifying the true model, regardless of the program used. This observation highlights the importance of carefully selecting the information criterion, with a preference for BIC, when determining the best-fit model for phylogenetic analyses. This study provides an assessment of popular model selection programs while contributing to the advancement of more robust statistical methods and tools for accurately identifying the most suitable nucleotide substitution models.

## Introduction

It is well known that nucleotide substitution models are widely used in phylogenetic analyses of sequence data, and distinct substitution models can change the outcome of phylogenetic analyses [1–3]. A nucleotide substitution model is a mathematical description of how DNA sequences change over time. It specifies the rates of substitution between all pairs of nucleotides, and the frequencies of each nucleotide in the sequence. A nucleotide substitution model can be simpler or more complex depending on how many parameters it has and how realistic it is. A simple model may assume that all substitutions are equally likely, and that all nucleotides have the same frequency. A complex model may allow for different rates of substitution for different types of changes (such as transitions and transversions), and for different frequencies of nucleotides depending on the context. A complex model may also account for variation in substitution rates among sites or among lineages [4–10]. Therefore, the selection of an appropriate substitution model is crucial for obtaining accurate phylogenetic inferences, as it directly influences the reliability of the resulting trees and downstream analyses [11–17].

In the last 20 years, a number of software for selecting the best-fit substitution model on a given dataset have been developed [18–22]. There are three statistical approaches to estimating how well a given substitution model fits a dataset, including the Akaike Information Criterion (AIC) [23], the Corrected Akaike Information Criterion (AICc) [24,25], both of them derived from frequentist probability, and the Bayesian Information Criterion (BIC) [26], which is derived from Bayesian probability. AIC [23], AICc [24,25] and BIC [26] are the most used model selection criteria and are implemented in a variety of softwares. BIC most heavily penalizes the addition of extra parameters, and substitution model selection parameters in turn. However, the results for selecting the best-fit model on a given dataset are not always consistent with one another, and the rule of thumb is that one should usually pick the model with smaller numbers of parameters for computational efficiency, expecially for a large dataset, when computing resources are limited [27]. Even though the selection of a simpler model might be preferable for computational efficiency, there are other points to be considered, such as the comparison of evolutionary rates among different genes/genomes/organisms, which are affected by the choice of substitution model applied to each dataset. jModelTest v2.1.10 [21,28], ModelTest-NG v0.1.7 [29,30] (ModelTest-NG is one to two orders of magnitude faster than jModelTest) [9], and IQ-TREE v2.2.0 [22,31] are some of the most popular software used for nucleotide substitution model selection, and all three have implemented AIC [23], AICc [24,25] and BIC [26] as model selection criteria.

Given the above, we sought to shed light on the following questions that molecular scientists frequently face. Are the statistical selection of best-fit models of nucleotide substitution by AIC [23] and AICc [24,25] consistent in most cases? If so, is statistical selection by AIC necessary when AICc has already been performed? When the best-fit model of nucleotide substitution selected by BIC [26] is inconsistent with that selected by AIC and AICc, should we use BIC or AIC/AICc? Furthermore, are the best-fit nucleotide substitution models selected by BIC usually simpler or more complex than those selected by AIC and AICc? If there is a difference, should we use the criterion that selects the best-fit nucleotide substitution models with fewer parameters? When the best-fit nucleotide substitution models selected irrespective of criteria in IQ-TREE are inconsistent with those selected in jModelTest2 or ModelTest-NG, which software results should be used? Furthermore, is the statistical selection by AIC, AICc, and BIC in jModelTest2 and ModelTest-NG usually simpler or more complex than those in IQ-TREE? If there is a difference, should we use the software that selects the best-fit nucleotide substitution model with fewer parameters? Notably, are the statistical selection of best-fit nucleotide substitution models by one criterion more frequently consistent with the real nucleotide substitution models used to generate simulated genetic datasets? If so, is statistical selection necessary when the former has already been performed?

While studies like Luo et al. [32], have explored this topic, a lack of consensus remains on which criteria or software should be prioritized in different modeling scenarios. Our study addresses this gap through a comprehensive comparative analysis. In summary, this study addresses the questions outlined above and provides insights into whether the selection of the best-fit nucleotide substitution models is influenced by the method and program used for implementation. If so, it suggests that, in certain cases, the selection of the best-fit nucleotide substitution model may lack objectivity.

## Materials and methods

To evaluate these questions, 34 published real datasets from a previous study [33] were investigated. These datasets contained multilocus DNA alignments from the mitochondrial, nuclear, and chloroplast genomes from a diverse array of animals and plants with a varying number of taxa (13 up to 2,872) and alignment lengths (823 up to 25,919 sites), providing a comprehensive representation of the diversity of genetic sequences used in phylogenetic studies. In addition, 88 published simulated datasets each generated with different nucleotide substitution models [34] were also investigated. These datasets contained 100 taxa with 10,000 nucleotides in length generated based on 88 random trees by AliSim software [34]. In summary, we analysed 122 datasets (34 real datasets and 88 simulated datasets) in the present study. For each dataset, the statistical selection of best-fit nucleotide substitution model by AIC, AICc, and BIC was performed in jModelTest v2.1.10 [21,28], ModelTest-NG v0.1.7 [29,30], and IQ-TREE v2.2.0 [22,31] using all substitution models offered in these software (S1 Table). The specific commands used for the statistical selection of the best-fit model are provided in S2 Table.

If different substitution models are selected using different criteria within the same software, we assess their similarity. Based on S3 Table, models that differ by four or fewer are considered similar, while those differing by five or more are deemed dissimilar (see S4 and S5 Tables). To evaluate the concordance between nucleotide substitution model selection results obtained from three different programs (using AIC, AICc, and BIC) and the true nucleotide substitution model, we conducted a statistical analysis. Specifically, we evaluated whether the best-fit models identified by each program and selection criterion were consistent with each other (S4 and S5 Tables) and with the known true model (S5 Table). This resulted in a binary classification (yes/no) reflecting agreement among the programs and with the true model (S1, S4, and S5 Tables, respectively). A Chi-squared test of independence was employed to determine if any significant associations existed between the programs, selection criteria, and the consistency of model selection. All statistical analyses were conducted in RStudio [35,36] using various packages, including ‘rcompanion’ [37], which provides the pairwiseNominalIndependence() function for post hoc analysis.

## Results

**Fig 1:**
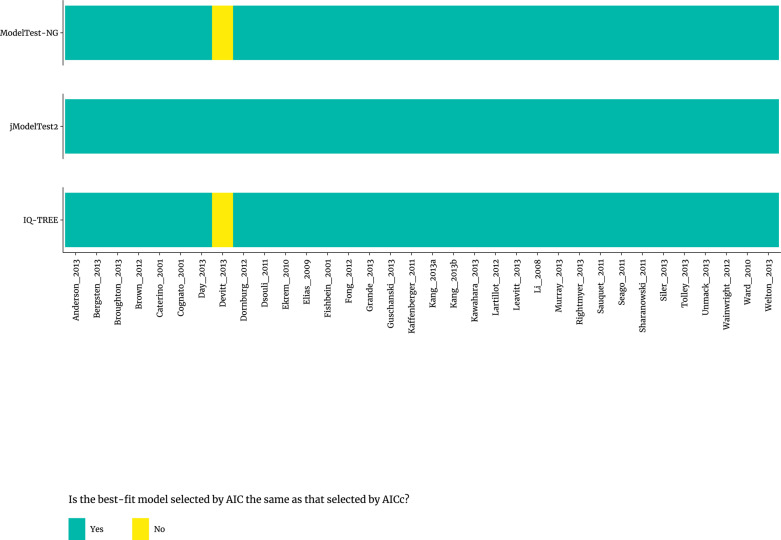
Results of statistical selection of best-fit models of nucleotide substitution by AIC in comparison to AICc using three different programs for real datasets.

S4 and S5 Tables present the statistical selection of best-fit nucleotide substitution model for the 34 published real datasets [33] and 88 published simulated datasets [34]. The model selection was performed using three different criteria—AIC, AICc, and BIC—and evaluated with three state-of-the-art programs: jModelTest2, ModelTest-NG, and IQ-TREE. For the 34 published real datasets [33], the best-fit nucleotide substitution model selected by AIC and AICc was the same in jModelTest2, ModelTest-NG and IQ-TREE, except for one dataset (‘Devitt_2013’) in ModelTest-NG and IQ-TREE (Fig 1). For the 88 published simulated datasets [34], as shown in S1 Fig selection by AIC and AICc was also the same in jModelTest2, ModelTest-NG and IQ-TREE, except for three datasets (‘HKY_F_I_G_10000’, ‘JC_I_G_10000’ and ‘TIM2e_10000’) in jModelTest2; one dataset (‘JC_G_10000’) in jModelTest2 and IQ-TREE.

**Fig 2:**
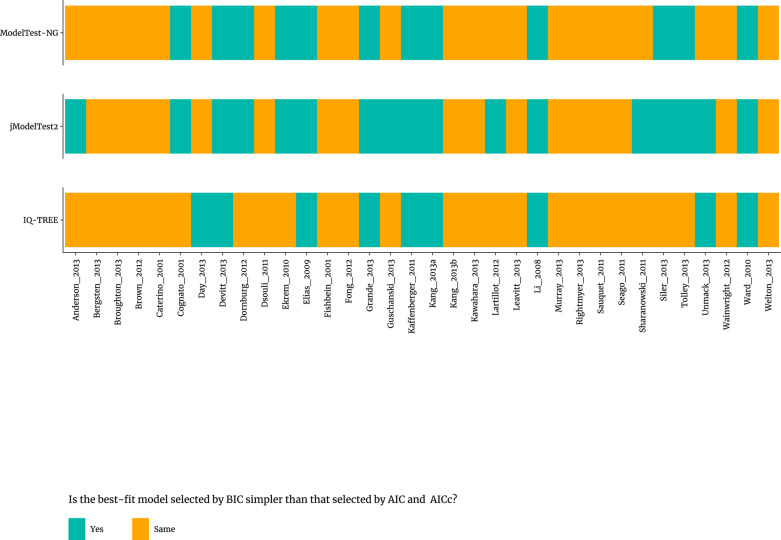
Results of statistical selection of best-fit models of nucleotide substitution by BIC in comparison to AIC and AICc using three different programs for real datasets.

Notably, for the 34 published real datasets [33] (Fig 2) and 88 published simulated datasets [34] (S2 Fig), when the selection of the best-fit models among methods was inconsistent, the best-fit models of nucleotide substitution selected by BIC were relatively simpler than those selected by AIC and AICc using the three different programs, except for one dataset (‘TVMe_10000’) in ModelTest-NG (S2 Fig).

**Fig 3:**
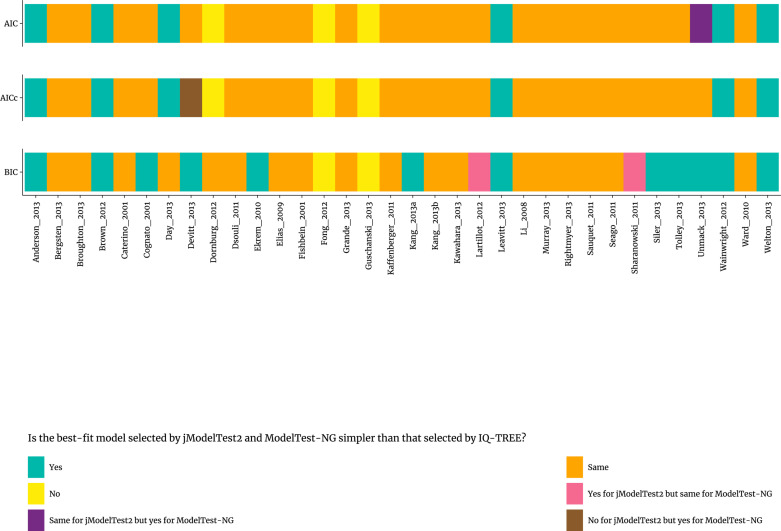
Results of statistical selection of best-fit models of nucleotide substitution by AIC, AICc, and BIC using jModelTest2 and ModelTest-NG in comparison to IQ-TREE for real datasets.

We also evaluated whether the best-fit nucleotide substitution models selected were consistent across software for each of the criteria. As shown in Fig 3 for the 34 published real datasets [33], when the best-fit nucleotide substitution models selected in jModelTest2 and ModelTest-NG were inconsistent with those selected in IQ-TREE, the statistical selection of the best-fit nucleotide substitution model selected by AIC performed in jModelTest2 and ModelTest-NG for six and seven datasets, respectively, prefered the relatively simpler models in comparison to the selection by AIC performed in IQ-TREE. However, the statistical selection of the best-fit nucleotide substitution model selected by AIC performed in IQ-TREE for three datasets tend to select relatively simpler models in comparison to the statistical selection by AIC performed both in jModelTest2 and ModelTest-NG. The statistical selection of the best-fit nucleotide substitution model selected by AICc performed in jModelTest2 and ModelTest-NG for six and seven datasets, respectively, prefers to select relatively simpler models in comparison to the selection by AICc performed in IQ-TREE, however, the statistical selection of the best-fit nucleotide substitution model selected by AICc performed in IQ-TREE for four and three datasets, respectively, tend to select relatively simpler models in comparison to the statistical selection by AICc performed in jModelTest2 and ModelTest-NG. For BIC, the statistical selection of the best-fit models of nucleotide substitution performed in jModelTest2 and ModelTest-NG for fourteen and twelve datasets, respectively, tends to select relatively simpler models in comparison to the statistical selection by BIC performed in IQ-TREE. However, the statistical selection of the best-fit nucleotide substitution model selected by BIC performed in IQ-TREE for two datasets tend to select relatively simpler models in comparison to the statistical selection by BIC performed both in jModelTest2 and ModelTest-NG.

For the 88 published simulated datasets [34], as shown in S3 Fig, when the best-fit models of nucleotide substitution selected by AIC, AICc, and BIC in jModelTest2 and ModelTest-NG were inconsistent with those selected in IQ-TREE, the statistical selection of the best-fit models of nucleotide substitution by AIC performed in jModelTest2 and ModelTest-NG for thirteen and sixteen datasets, respectively, tends to select relatively simpler models in comparison to the statistical selection by AIC performed in IQ-TREE. However, the statistical selection of the best-fit models of nucleotide substitution by AIC performed in IQ-TREE for ten and eight datasets, respectively, leans towards selection of relatively simpler models in comparison to the statistical selection by AIC performed in jModelTest2 and ModelTest-NG. Similarly, for AICc, the statistical selection of the best-fit models of nucleotide substitution performed in jModelTest2 and ModelTest-NG for twelve and fifteen datasets, respectively, tends to select relatively simpler models in comparison to the statistical selection by AICc performed in IQ-TREE. But again, there are instances (eight datasets) where the statistical selection of the best-fit models of nucleotide substitution by AICc performed in IQ-TREE tends to select relatively simpler models in comparison to the statistical selection by AICc performed both in jModelTest2 and ModelTest-NG. Lastly, for BIC, the statistical selection of the best-fit models of nucleotide substitution performed in jModelTest2 and ModelTest-NG for just one dataset gravitates towards selection of relatively simpler models in comparison to the statistical selection by BIC performed in IQ-TREE. Interestingly, the statistical selection of the best-fit models of nucleotide substitution by BIC performed in IQ-TREE never selects a relatively simpler model in comparison to the statistical selection by BIC performed both in jModelTest2 and ModelTest-NG.

**Fig 4:**
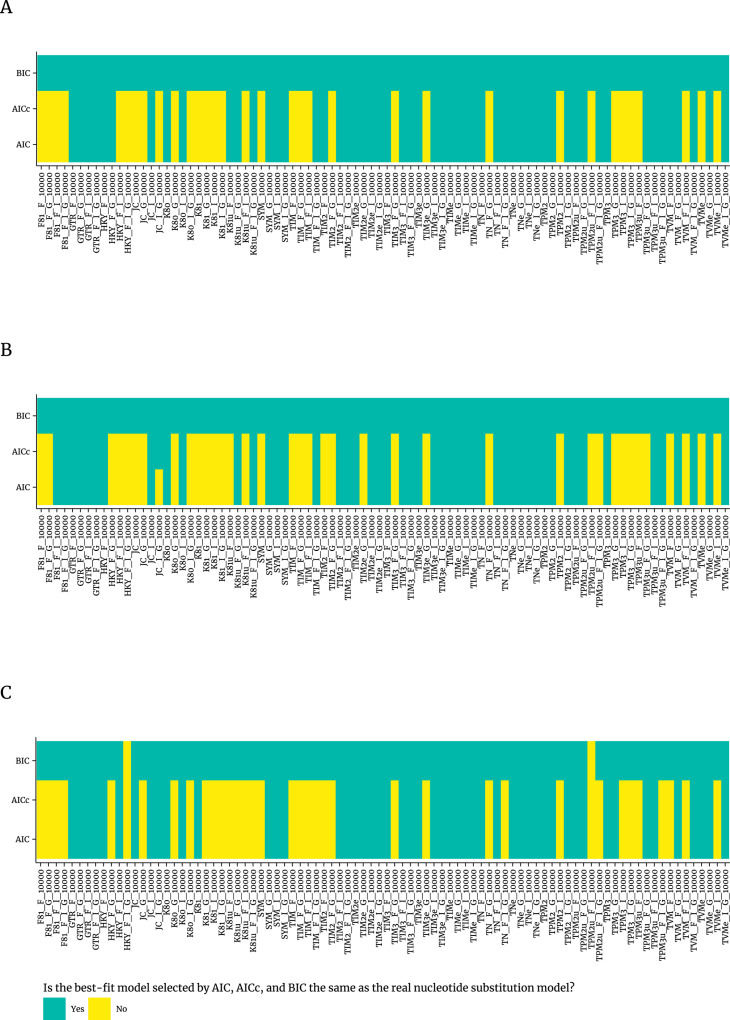
Results of statistical selection of best-fit models of nucleotide substitution by AIC, AICc, and BIC using three different programs in comparison to real nucleotide substitution model for simulated datasets.

The results of statistical selection of best-fit models of nucleotide substitution by AIC, AICc, and BIC using three different programs in comparison to real nucleotide substitution model for the 88 published simulated datasets [34] are shown in Fig 4. The statistical selection of the best-fit models of nucleotide substitution by AIC performed in jModelTest2, ModelTest-NG, and IQ-TREE for 50 (50/88; 56.8%), 55 (55/88; 62.5%) and 51(51/88; 58.0%) datasets, respectively, were consistent with real nucleotide substitution models. Similarly and as expected, the statistical selection of the best-fit models of nucleotide substitution by AICc performed in jModelTest2, ModelTest-NG, and IQ-TREE for 51(51/88; 58.0%), 55 (55/88; 62.5%) and 51(51/88; 58.0%) datasets, respectively, were consistent with real nucleotide substitution models, and thus performed better than the previous criterion. Remarkably, the statistical selection of the best-fit models of nucleotide substitution by BIC performed in jModelTest2, ModelTest-NG, and IQ-TREE for 88 (88/88; 100%), 88 (88/88; 100%) and 86 (86/88; 97.7%) datasets, respectively, were consistent with real nucleotide substitution models.

Using AIC, AICc, and BIC criteria, for the 34 published real datasets [33], as shown in S4 Tables, the proportion of nucleotide substitution best model similarities detected by jModelTest2, ModelTest-NG, and IQ-TREE are 19/34, 24/34, and 26/34, respectively. In jModelTest2, the nucleotide substitution best model similarities in 19 out of 34 datasets were also detected by ModelTest-NG. ModelTest-NG detected the nucleotide substitution best model similarity in 5 additional datasets compared to jModelTest2. There are 5 datasets where the nucleotide substitution best model similarity detection results differ between ModelTest-NG and jModelTest2. There are 8 datasets where the nucleotide substitution best model similarity detection results differ between ModelTest-NG and IQ-TREE. The nucleotide substitution best model similarity detection results differ between ModelTest-NG, jModelTest2, and IQ-TREE for 11 datasets.

Using AIC, AICc, and BIC criteria, for the 88 published simulated datasets [34], as shown in S5 Table, the proportion of nucleotide substitution best model similarities detected by jModelTest2, ModelTest-NG, and IQ-TREE are 67/88, 71/88, and 64/88, respectively. In jModelTest2, the nucleotide substitution best model similarities in 67 out of 88 datasets were also detected by ModelTest-NG. ModelTest-NG detected the nucleotide substitution best model similarity in 4 additional datasets compared to jModelTest2. There are 4 datasets where the nucleotide substitution best model similarity detection results differ between ModelTest-NG and jModelTest2. There are 13 datasets where the nucleotide substitution best model similarity detection results differ between ModelTest-NG and IQ-TREE. The nucleotide substitution best model similarity detection results differ between ModelTest-NG, jModelTest2, and IQ-TREE for 13 datasets.

To assess the consistency of model selection across three different programs (jModelTest2, ModelTest-NG, and IQ-TREE), we evaluated their ability to identify the true nucleotide substitution model. Each program was used to select the best-fit model based on three information criteria (AIC, AICc, and BIC) (S5 Table). The results, summarized in Table 1, show the number of instances (irrespective of information criteria) where each program successfully identified the true model.

**Table 1:** Frequency of correctly identifying the true nucleotide substitution model.

Program	Yes	No
jModelTest2	189	75
ModelTest-NG	198	66
IQ_TREE	188	76

A Chi-squared test of independence was performed to determine if any significant differences existed in the accuracy of model selection among the three programs. Pairwise comparisons, presented in Table 2, revealed no significant differences (all adjusted p-values >  0.05). This suggests that the choice of program does not significantly impact the ability to identify the true nucleotide substitution model.

**Table 2:** Pairwise comparisons of model selection accuracy between programs.

Comparison	p.Chisq	p.adj.Chisq	Cramer.V
IQ_TREE: jModelTest2	0.92	0.92	0.00
IQ_TREE: ModelTest-NG	0.33	0.56	0.04
jModelTest2: ModelTest-NG	0.38	0.56	0.04

To assess the consistency of model selection across three different information criteria (AIC, AICc, and BIC) (S5 Table), we evaluated their ability to identify the true nucleotide substitution model across datasets. The results, summarized in Table 3, show the number of instances where each criterion successfully identified the true model.

**Table 3:** Frequency of correctly identifying the true nucleotide substitution model using different information criteria.

Criteria	Yes	No
AIC	156	108
AICc	157	107
BIC	262	2

A Chi-squared test of independence was performed to determine if any significant differences existed in the accuracy of model selection among the three criteria. The overall test was statistically significant (χ^2^ = 141.31, df =  2, p <  2.2 x 10^-16^), indicating that the choice of information criterion significantly impacts the ability to identify the true model.

Pairwise comparisons, presented in Table 4, were conducted post hoc to identify the source of these differences. Each pairwise comparison was also statistically significant (all adjusted p-values <  0.05), indicating that AIC, AICc, and BIC each differ significantly in their ability to select the true model. Notably, BIC demonstrated a substantially higher accuracy compared to both AIC and AICc.

**Table 4:** Pairwise comparisons of model selection accuracy between information criteria.

Comparison	p.Chisq	p.adj.Chisq	Cramer.V
AIC: AICc	9.29 x 10^-1^	9.29 x 10^-1^	0.00
AIC: BIC	6.69 x 10^-30^	2.01 x 10^-29^	0.49
AICc: BIC	1.47 x 10^-29^	2.20 x 10^-29^	0.49

These findings highlight the importance of carefully considering the choice of information criterion for model selection in phylogenetic analyses. While AIC and AICc produced similar results, BIC demonstrated a clear advantage in identifying the true nucleotide substitution model.

To assess the influence of information criteria on model selection across different programs, we evaluated the performance of AIC, AICc, and BIC in jModelTest2, ModelTest-NG, and IQ-TREE. Each program was used to select the best-fit nucleotide substitution model for 88 datasets (S5 Table), and the frequency of correctly identifying the true model was recorded (Tables 5–10).

**Table 5:** Frequency of correctly identifying the true nucleotide substitution model using different information criteria in jModelTest2.

Criteria	Yes	No
AIC	50	38
AICc	51	37
BIC	88	0

**Table 6:** Pairwise comparisons of model selection accuracy in jModelTest2 using different information criteria.

Comparison	p.Chisq	p.adj.Chisq	Cramer.V
AIC: AICc	8.79 x 10^-1^	8.79 x 10^-1^	0.01
AIC: BIC	3.36 x 10^-12^	1.01 x 10^-11^	0.53
AICc: BIC	7.67 x 10^-12^	1.15 x 10^-11^	0.52

**Table 7:** Frequency of correctly identifying the true nucleotide substitution model using different information criteria in ModelTest-NG.

Criteria	Yes	No
AIC	55	33
AICc	55	33
BIC	88	0

**Table 8:** Pairwise comparisons of model selection accuracy in ModelTest-NG using different information criteria.

Comparison	p.Chisq	p.adj.Chisq	Cramer.V
AIC: AICc	1.00	1.00	0.00
AIC: BIC	1.85 x 10^-10^	2.78 x 10^-10^	0.48
AICc: BIC	1.85 x 10-10	2.78 x 10^-10^	0.48

**Table 9:** Frequency of correctly identifying the true nucleotide substitution model using different information criteria in IQ-TREE.

Criteria	Yes	No
AIC	51	37
AICc	51	37
BIC	86	2

**Table 10:** Pairwise comparisons of model selection accuracy in IQ-TREE using different information criteria.

Comparison	p.Chisq	p.adj.Chisq	Cramer.V
AIC: AICc	1.00	1.00	0.00
AIC: BIC	2.12 x 10^-10^	3.18 x 10^-10^	0.48
AICc: BIC	2.12 x 10^-10^	3.18 x 10^-10^	0.48

A Chi-squared test of independence revealed significant differences in the accuracy of model selection among the three information criteria in jModelTest2 (χ^2^ =  52.409, df =  2, p <  4.164 x 10^-12^) (Table 5). Pairwise comparisons (Table 6) showed that BIC significantly outperformed both AIC and AICc (adjusted p <  0.05), while there was no significant difference between AIC and AICc.

Similarly, in ModelTest-NG, a significant difference was observed among the criteria (χ^2^ =  44, df =  2, p <  2.789 x 10^-10^) (Table 7). Again, BIC showed significantly higher accuracy compared to both AIC and AICc (adjusted p <  0.05), with no significant difference between AIC and AICc (Table 8).

In IQ-TREE, the pattern remained consistent. The overall Chi-squared test was significant (χ^2^ =  45.269, df =  2, p <  1.479 x 10^-10^) (Table 9), and BIC was significantly more accurate than both AIC and AICc (adjusted p <  0.05), with no difference between AIC and AICc (Table 10).

To assess the consistency of model selection across different programs, we evaluated the agreement between jModelTest2, ModelTest-NG, and IQ-TREE in identifying the best-fit nucleotide substitution model. Each program was used to select the best model based on three information criteria (AIC, AICc, and BIC) for the 34 real datasets (S4 Table). We then compared whether the models selected by each program were identical across all three criteria, resulting in a binary classification (yes/no) for each program (Table 11).

**Table 11:** Frequency of consistent model selection across different information criteria for each program.

Program	Similarity of nucleotide substitution model	
	Yes	No
jModelTest2	19	15
ModelTest-NG	24	10
IQ_TREE	26	8

A Chi-squared test of independence was performed to determine if any significant differences existed in the consistency of model selection among the three programs. The test was not statistically significant (χ^2^ =  3.4941, df =  2, p =  0.1743), indicating that the choice of program does not significantly impact the agreement in model selection across different information criteria. This suggests that the three programs generally produce similar results when selecting the best-fit model, regardless of the specific criterion used.

To assess the consistency of model selection across different programs using simulated datasets, we evaluated the agreement between jModelTest2, ModelTest-NG, and IQ-TREE in identifying the best-fit nucleotide substitution model. Each program was used to select the best model based on three information criteria (AIC, AICc, and BIC) for 88 simulated datasets (S5 Table). We then compared whether the models selected by each program were identical across all three criteria, resulting in a binary classification (yes/no) for each program (Table 12).

**Table 12:** Frequency of consistent model selection across different information criteria for each program using simulated datasets.

Program	Similarity of nucleotide substitution model	
	Yes	No
jModelTest2	67	21
ModelTest-NG	71	17
IQ_TREE	64	24

A Chi-squared test of independence was performed to determine if any significant differences existed in the consistency of model selection among the three programs. The test was not statistically significant (χ^2^ =  1.5599, df =  2, p =  0.4584), indicating that the choice of program does not significantly impact the agreement in model selection across different information criteria when using simulated data. This suggests that, similar to the results observed with real datasets, the three programs generally produce similar results when selecting the best-fit model from simulated data, regardless of the specific criterion used.

## Discussion

The statistical selection of best-fit models of nucleotide substitution for multiple sequence alignments (MSAs) of DNA or RNA is routine in phylogenetics [38]. Previous study has shown that BIC is preferred for nucleotide substitution of molecular evolutionary genetic analysis in a comprehensive study [32]. In the present study, we investigated the general principles for statistical selection of best-fit models of nucleotide substitution using 122 published datasets (34 real datasets [33] and 88 simulated datasets [34]), using three selection methods (AIC, AICc, and BIC) and three state-of-the-art programs (jModelTest2, ModelTest-NG, and IQ-TREE). Our finding showed that model selections by AIC and AICc were the same in most cases for both the 34 published real datasets [33] and 88 published simulated datasets [34] (Figs 1 and S1). We observed that, when model selection was inconsistent across methods, the nucleotide substitution models selected by BIC were generally simpler than those chosen by AIC and AICc. This pattern was consistent across all 34 real [33] and 88 simulated datasets [34], except for one dataset (‘TVMe_10000’) in ModelTest-NG, using three different software programs (Figs 2 and S2). Additionally, though evolution is often a complex process, for computational purposes, researcher tend to select the simplest model that can appropriately characterize the evolutionary process [39]. This is in line with similar comparisons in the context of machine learning (https://machinelearningmastery.com/probabilistic-model-selection-measures).

Second, when best-fit model selection was inconsistent among different programs, AIC, AICc, and BIC tended to select relatively simpler best-fit models of nucleotide substitution in jModelTest2 and ModelTest-NG than in IQ-TREE in most cases for both the 34 published real datasets [33] and 88 published simulated datasets [34], especially, for BIC method (Figs 3 and S3). Notably, the statistical selection of the best-fit models of nucleotide substitution by BIC performed in jModelTest2, ModelTest-NG, and IQ-TREE were much more often consistent (100%, 100%, and 97.7%, respectively) with the real nucleotide substitution models of simulated datasets [34] (Fig 4) in comparision to those obtained using AIC (56.8%, 62.5%, 58.0%, respectively) or AICc (58.0%, 62.5%, 58.0%, respectively). We compared the performance of jModelTest2, ModelTest-NG, and IQ-TREE in selecting nucleotide substitution models using AIC, AICc, and BIC criteria across the 34 published real datasets [33] and 88 published simulated datasets [34] (S4 and S5 Tables). The performance of different programs in selecting the best-fit nucleotide substitution model can vary due to several key factors: algorithmic approach, selection criteria, model variety, handling complexity, computational efficiency, ease of use, and software updates/support [21,22,28,29,31,40,41]. Though not statistically significant, ModelTest-NG was often more reliable and accurate in selecting the best-fit nucleotide substitution model. It combines modern algorithms with a scientifically robust methodology to ensure that the selected models are both statistically sound and generalizable, making it the optimal choice for model selection in molecular evolutionary analyses.

Our analysis of model selection accuracy across three popular phylogenetic programs (jModelTest2, ModelTest-NG, and IQ-TREE) revealed that the choice of program had no significant impact on the ability to identify the true nucleotide substitution model. This finding suggests that researchers can confidently use any of these programs for model selection without concern for substantial differences in accuracy. However, in agreement with previous studies [32], our results did highlight the critical influence of the information criterion used for model selection. BIC consistently outperformed both AIC and AICc in identifying the true model, irrespective of the program employed. This observation underscores the importance of carefully considering the information criterion, and potentially favoring BIC, when selecting the best-fit model for phylogenetic analyses. While further research is needed to explore the generalizability of these findings across diverse datasets and evolutionary scenarios, our results provide valuable insights for researchers seeking to optimize model selection strategies in phylogenetics.

Other limitations include only testing 88 substitution models as per the 88 simulated datasets, however, jModelTest2 can test 1624 substitution models and IQ-TREE can test an even higher number of substitution models than jModelTest2. However, the substitution models studied here are a good representation of those implemented in the most popular phylogenetic tree reconstruction software (*i.e.*, MEGA [42], FastTree [43], PhyML [44], RAxML [41], RAxML-NG [45], IQ-TREE [31], MrBayes [46], BEAST [47,48]). We did not test the substitution model selection using the famous MEGA software because it only supports 24 substitution models. The 88 published simulated datasets, each generated with different nucleotide substitution models, were tested using the three state-of-the-art programs (jModelTest2, ModelTest-NG, and IQ-TREE) for comparison, one of which defaults settings only allow testing 88 substitution models (ModelTest-NG).

While previous studies [32] have explored this topic, clear guidance on prioritizing specific criteria or software for different modeling scenarios remains lacking. Our study fills this gap with a comprehensive comparative analysis to resolve these uncertainties. Overall, our results indicate that the selection methods employed by different programs influence the choice of the best-fit nucleotide substitution model. Based on a comprehensive statistical analysis of these patterns, we recommend using the Bayesian Information Criterion (BIC) implemented in most softwares for the statistical selection of the best-fit nucleotide substitution model. We hope that this study will contribute to the development of more robust statistical selection methods and tools for accurately identifying the most appropriate nucleotide substitution models.

## Supporting information

S1 Fig Results of statistical selection of best-fit models of nucleotide substitution by AIC in comparison to AICc using three different programs for simulated datasets. (PDF)

S2 Fig Results of statistical selection of best-fit models of nucleotide substitution by BIC in comparison to AIC and AICc using three different programs for simulated datasets. (PDF)

S3 Fig Results of statistical selection of best-fit models of nucleotide substitution by AIC, AICc, and BIC using jModelTest2 and ModelTest-NG in comparison to IQ-TREE for simulated datasets. (PDF)

S1 Table List of the 88 nucleotide substitution models sorted by ModelTest-NG. (XLSX)

S2 Table Specific command lines used for statistical selection of best-fit models of nucleotide substitution. (XLSX)

S3 Table All common DNA susbtitution models (ordered by complexity). Adapted from http://www.iqtree.org/doc/Substitution-Models on December 11, 2024.
(XLSX)

S4 Table Results of statistical selection of best-fit models of nucleotide substitution by AIC, AICc, and BIC using three different programs for real datasets. (XLSX)

S5 Table Results of statistical selection of best-fit models of nucleotide substitution by AIC, AICc, and BIC using three different programs for simulated datasets. (XLSX)
