# Supplementary figures and images for: The impact of software and criteria on the selection of best-fit nucleotide substitution models for molecular evolutionary genetic analysis
Source: PLoS One. 2025 Mar 26;20(3):e0319774. doi: 10.1371/journal.pone.0319774 (PMC11940733; doi:10.1371/journal.pone.0319774)

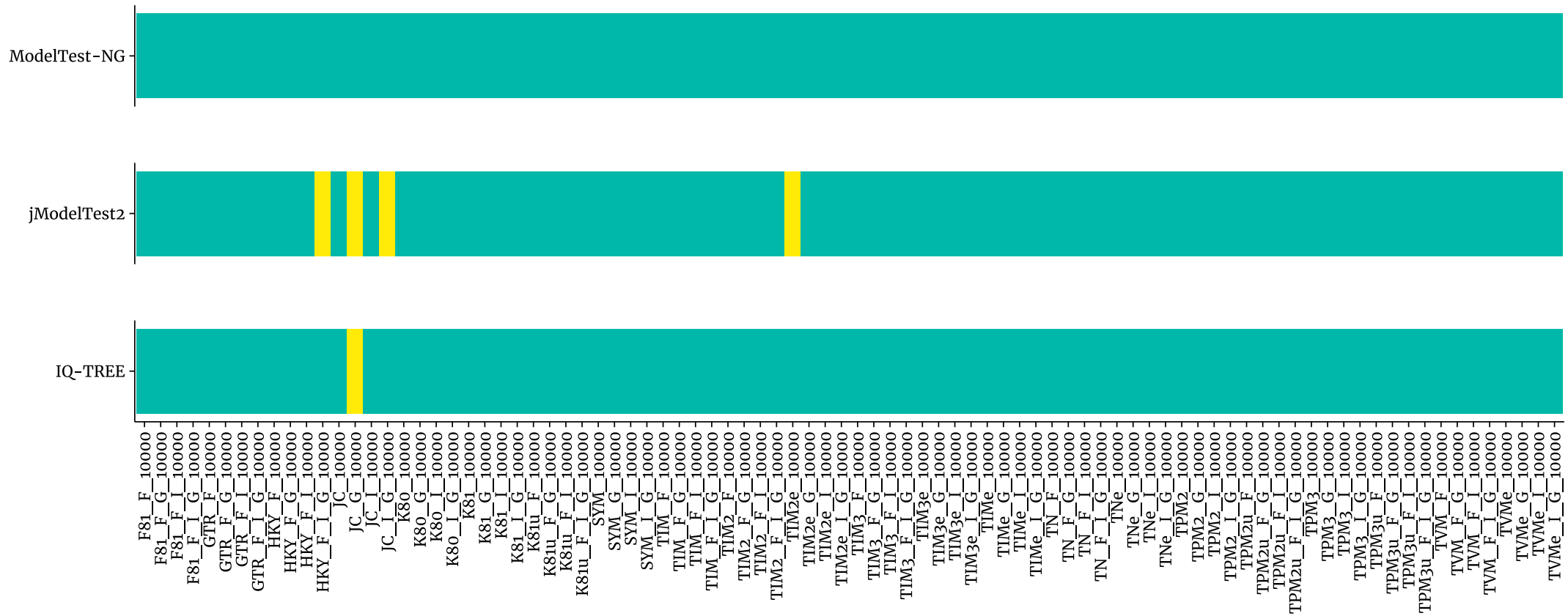

Supplement: S1 Fig — (PDF) [file pone.0319774.s001.pdf]

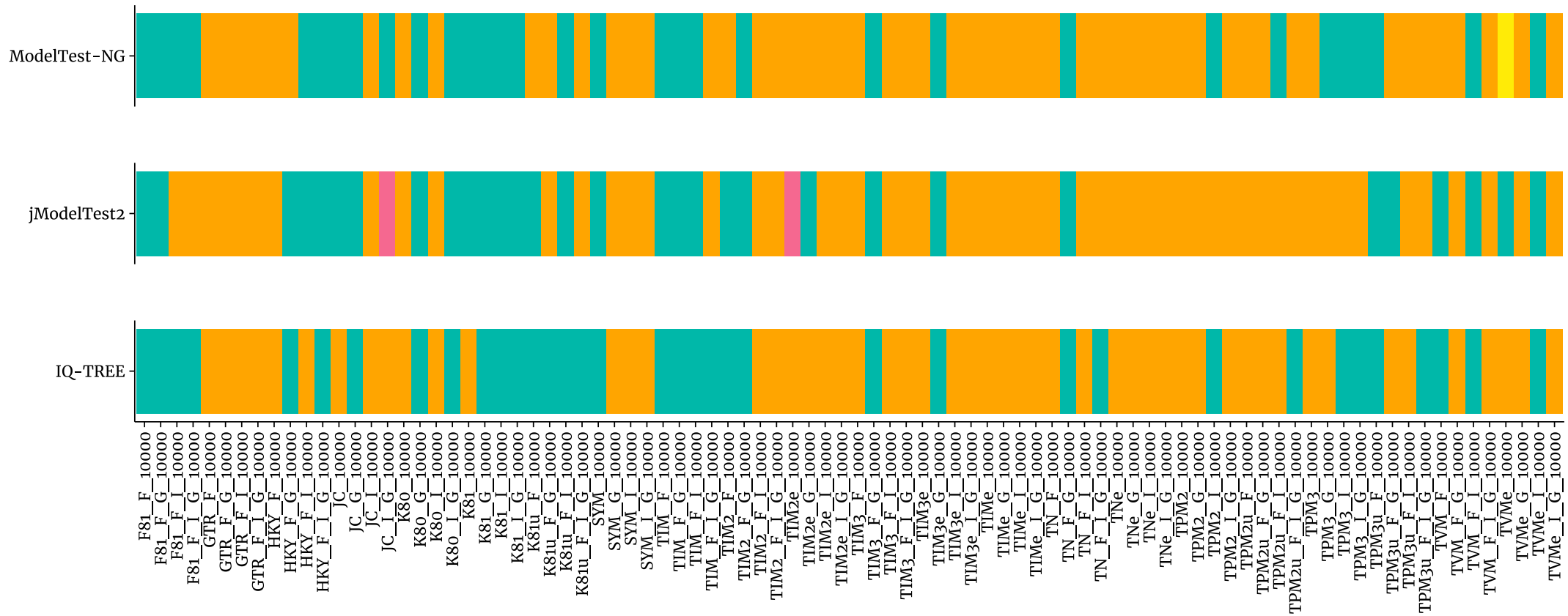

Is the best-fit model selected by BIC simpler than that selected by AIC and AICc?

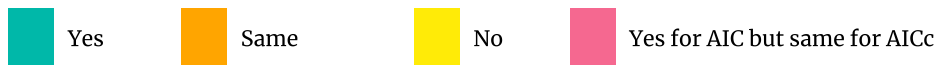

Supplement: S2 Fig — (PDF) [file pone.0319774.s002.pdf]
