# Supplementary material for: The impact of software and criteria on the selection of best-fit nucleotide substitution models for molecular evolutionary genetic analysis
Source: PLoS One. 2025 Mar 26;20(3):e0319774. doi: 10.1371/journal.pone.0319774 (PMC11940733; doi:10.1371/journal.pone.0319774)
Supplement: S3 Fig — (PDF) [file pone.0319774.s003.pdf]

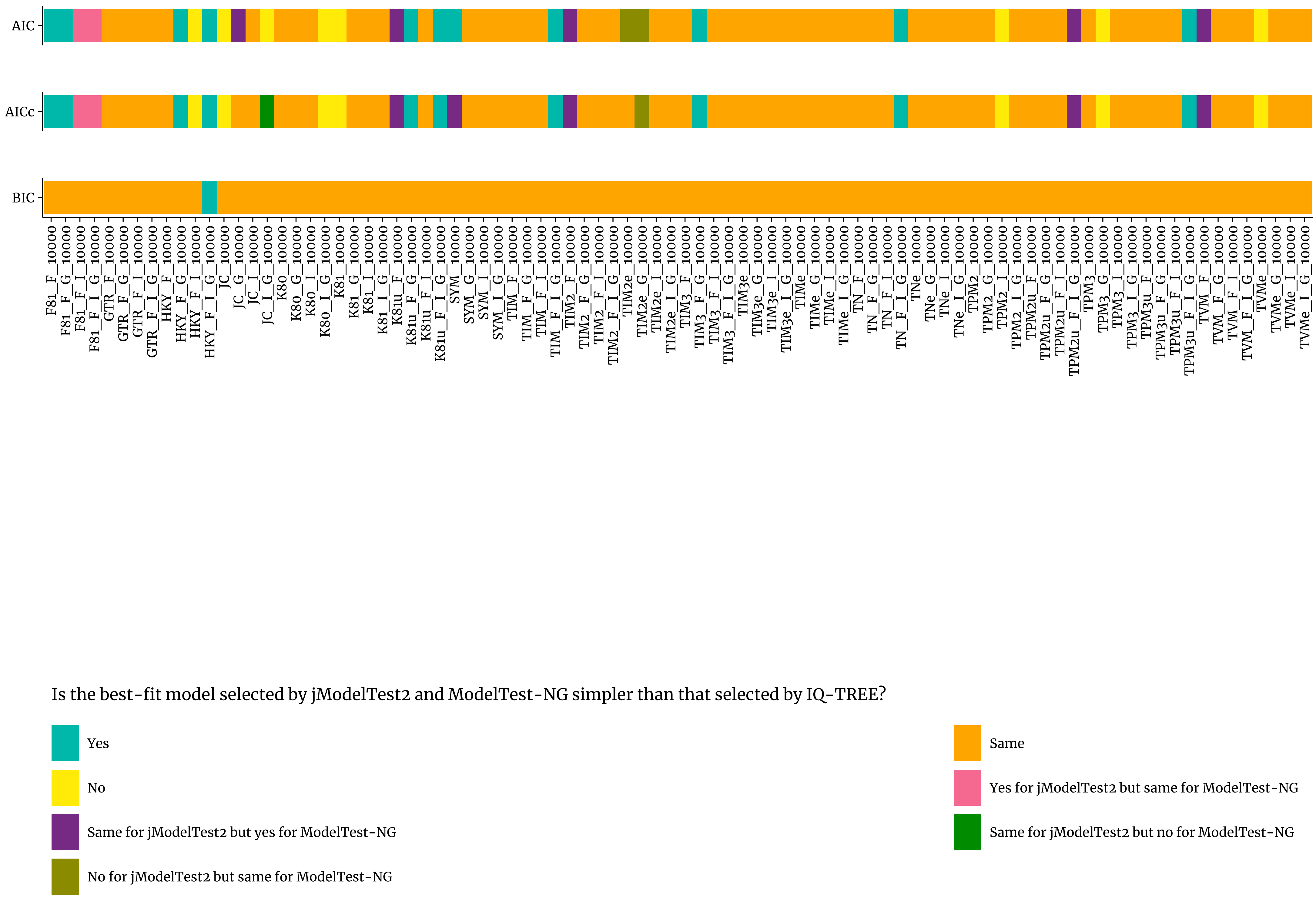
AIC
AICc
BIC
F81_F_10000
F81_F_G_10000
F81_F_I_10000
F81_F_I_G_10000
GTR_F_10000
GTR_F_G_10000
GTR_F_I_10000
GTR_F_I_G_10000
HKY_F_10000
HKY_F_G_10000
HKY_F_I_10000
HKY_F_I_G_10000
JC_10000
JC_G_10000
JC_I_10000
JC_I_G_10000
K80_10000
K80_G_10000
K80_I_10000
K80_I_G_10000
K81_10000
K81_G_10000
K81_I_10000
K81_I_G_10000
K81u_F_10000
K81u_F_G_10000
K81u_F_I_10000
K81u_F_I_G_10000
SYM_10000
SYM_G_10000
SYM_I_10000
SYM_I_G_10000
TIM_F_10000
TIM_F_G_10000
TIM_F_I_10000
TIM_F_I_G_10000
TIM2_F_10000
TIM2_F_G_10000
TIM2_F_I_10000
TIM2_F_I_G_10000
TIM2e_10000
TIM2e_G_10000
TIM2e_I_10000
TIM2e_I_G_10000
TIM3_F_10000
TIM3_F_G_10000
TIM3_F_I_10000
TIM3_F_I_G_10000
TIM3e_10000
TIM3e_G_10000
TIM3e_I_10000
TIM3e_I_G_10000
TIMe_10000
TIMe_G_10000
TIMe_I_10000
TIMe_I_G_10000
TN_F_10000
TN_F_G_10000
TN_F_I_10000
TN_F_I_G_10000
TNe_10000
TNe_G_10000
TNe_I_10000
TNe_I_G_10000
TPM2_10000
TPM2_G_10000
TPM2_I_10000
TPM2_I_G_10000
TPM2u_F_10000
TPM2u_F_G_10000
TPM2u_F_I_10000
TPM2u_F_I_G_10000
TPM3_10000
TPM3_G_10000
TPM3_I_10000
TPM3_I_G_10000
TPM3u_F_10000
TPM3u_F_G_10000
TPM3u_F_I_10000
TPM3u_F_I_G_10000
TVM_F_10000
TVM_F_G_10000
TVM_F_I_10000
TVM_F_I_G_10000
TVMe_10000
TVMe_G_10000
TVMe_I_10000
TVMe_I_G_10000
Is the best-fit model selected by jModelTest2 and ModelTest-NG simpler than that selected by IQ-TREE?
Yes
No
Same for jModelTest2 but yes for ModelTest-NG
No for jModelTest2 but same for ModelTest-NG
Same
Yes for jModelTest2 but same for ModelTest-NG
Same for jModelTest2 but no for ModelTest-NG
